# Using Continuous Glucose Monitoring to Detect and Intervene on Dietary Restriction in Individuals With Binge Eating: The SenseSupport Withdrawal Design Study

**DOI:** 10.2196/38479

**Published:** 2022-12-14

**Authors:** Adrienne S Juarascio, Paakhi Srivastava, Emily K Presseller, Mandy Lin, Anna G G Patarinski, Stephanie M Manasse, Evan M Forman

**Affiliations:** 1 Center for Weight, Eating, and Lifestyle Science Drexel University Philadelphia, PA United States; 2 Department of Psychological and Brain Sciences Drexel University Philadelphia, PA United States

**Keywords:** binge eating, loss-of-control eating, continuous glucose monitoring, mobile phone

## Abstract

**Background:**

Dietary restraint is a key factor for maintaining engagement in binge eating among individuals with binge eating disorder (BED) and bulimia nervosa (BN). Reducing dietary restraint is a mechanism of change in cognitive behavioral therapy (CBT) for individuals with BN and BED. However, many individuals who undergo CBT fail to adequately reduce dietary restraint during treatment, perhaps owing to difficulty in using treatment skills (eg, regular eating) to reduce dietary restraint during their daily lives. The SenseSupport system, a novel just-in-time, adaptive intervention (JITAI) system that uses continuous glucose monitoring to detect periods of dietary restraint, may improve CBT to reduce dietary restraint during treatment by providing real-time interventions.

**Objective:**

This study aimed to describe the feasibility, acceptability, and initial evaluation of SenseSupport. We presented feasibility, acceptability, target engagement, and initial treatment outcome data from a small trial using an ABAB (A=continuous glucose monitoring data sharing and JITAIs-Off, B=continuous glucose monitoring data sharing and JITAIs-On) design (in which JITAIs were turned on for 2 weeks and then turned off for 2 weeks throughout the treatment).

**Methods:**

Participants (N=30) were individuals with BED or BN engaging in ≥3 episodes of ≥5 hours without eating per week at baseline. Participants received 12 sessions of CBT and wore continuous glucose monitors to detect eating behaviors and inform the delivery of JITAIs. Participants completed 4 assessments and reported eating disorder behaviors, dietary restraint, and barriers to app use weekly throughout treatment.

**Results:**

Retention was high (25/30, 83% after treatment). However, the rates of continuous glucose monitoring data collection were low (67.4% of expected glucose data were collected), and therapists and participants reported frequent app-related issues. Participants reported that the SenseSupport system was comfortable, minimally disruptive, and easy to use. The only form of dietary restraint that decreased significantly more rapidly during JITAIs-On periods relative to JITAIs-Off periods was the desire for an empty stomach (*t*_43_=1.69; *P*=.049; Cohen *d*=0.25). There was also a trend toward greater decrease in overall restraint during JITAs-On periods compared with JITAIs-Off periods, but these results were not statistically significant (*t*_43_=1.60; *P*=.06; Cohen *d*=0.24). There was no significant difference in change in the frequency of binge eating during JITAIs-On periods compared with JITAIs-Off periods (*P*=.23). Participants demonstrated clinically significant, large decreases in binge eating (*t*_24_=10.36; *P*<.001; Cohen *d*=2.07), compensatory behaviors (*t*_24_=3.40; *P*=.001; Cohen *d*=0.68), and global eating pathology (*t*_24_=6.25; *P*<.001; Cohen *d*=1.25) from pre- to posttreatment.

**Conclusions:**

This study describes the successful development and implementation of the first intervention system combining passive continuous glucose monitors and JITAIs to augment CBT for binge-spectrum eating disorders. Despite the lower-than-anticipated collection of glucose data, the high acceptability and promising treatment outcomes suggest that the SenseSupport system warrants additional investigation via future, fully powered clinical trials.

**Trial Registration:**

ClinicalTrials.gov NCT04126694; https://clinicaltrials.gov/ct2/show/NCT04126694

## Introduction

### Background

Binge eating (ie, eating a large amount of food within a discrete time period accompanied by a sense of loss of control overeating) is a key symptom of several eating disorders (EDs), including bulimia nervosa (BN) and binge ED (BED). According to a recent study using a nationally representative sample, as many as 3.7 million Americans will have a lifetime BN or BED diagnosis [1] and as many as 42.2 million Americans will experience clinically significant binge eating [2]. EDs are considered critical public health issues, are associated with significant negative physical and psychosocial consequences [3-10], and place a substantial burden on health care services [11].

Dietary restriction (ie, deliberate attempts to drastically reduce the overall amount of food eaten or the types of food eaten) is a key maintenance factor for binge eating in BN and BED, and reducing dietary restriction is the most well-established mechanism of existing treatments. Dietary restriction increases the vulnerability to binge eating episodes because it leaves patients in a state of physical or psychological deprivation or both [12]. Moreover, reducing dietary restriction is one of the only treatment mechanisms that has been empirically supported [13-19]. Furthermore, the adoption of a regular eating schedule (eg, eating 3 meals and 1 or 2 snacks per day and not going >4 waking hours without eating) is one of the strongest predictors of treatment success for both BN and BED [14,20-22]. Thus, dietary restriction has been identified as an essential clinical target for the treatment of BN and BED.

Cognitive behavioral therapy (CBT), including an enhanced transdiagnostic version, is the current frontline treatment approach for both BN and BED [23-25]. Most CBT manuals focus >50% of the session content on the reduction of dietary restriction and recommend achieving a regular eating schedule before moving on to other treatment content [25-27]. However, many patients with BN and BED continue to engage in restrictive eating behaviors until the end of treatment, suggesting that CBT fails to sufficiently improve this clinical target [28]. For example, one trial of CBT found that <40% of participants achieved regular eating (defined as eating at least three meals and one snack per day) in the month immediately before the posttreatment assessment [20]. In addition, recent systematic reviews and meta-analyses have found that 40% to 50% of patients with BED [29] and nearly 70% of patients with BN [13] remain symptomatic after a full course of CBT, likely owing in part to insufficient amelioration of dietary restriction.

CBT may fail to adequately improve dietary restriction because the typical method of intervention delivery (eg, weekly in-person therapy sessions) limits the ability to intervene during the moments when an intervention may be most needed. The inability of CBT to intervene sufficiently in dietary restriction may be due to 3 main reasons. First, dietary restriction can fluctuate significantly within a day and between days in individuals with BN and BED [30]. For example, within any given day, restrictive eating behaviors can re-emerge following binge eating episodes, as individuals attempt to control weight via restriction [25]. Indeed, research has shown that individuals with BN and BED often engage in *chaotic* eating, ie, profound within- and between-day fluctuations in meal and calorie patterning [31]. Given the within- and between-day fluctuations in restrictive intent and behavior, it is not surprising that weekly treatment approaches may fail to alter this constantly moving target. Second, CBT relies on patients’ self-reporting (typically through a review of self-monitoring records that involves tracking eating and ED behaviors) to identify continued engagement in restrictive eating behaviors. Even among individuals without eating pathologies, accurate tracking of dietary intake is notoriously difficult [32-34]. In patients with eating pathology, many individuals are unable or unwilling to accurately record their eating behaviors, including restrictive eating, which limits a CBT clinician’s ability to intervene appropriately to prevent dietary restriction. Finally, obtaining adequate compliance with self-monitoring records during CBT can be difficult because of the perceived burden during a long-term treatment [14]. Thus, the limitations in both the quality of data used to guide interventions and the frequency and immediacy with which interventions can be provided may explain why CBT results in inadequate amelioration of dietary restriction.

### Objectives

The limitations described above for traditional in-person CBT suggest that patients may need additional real-time support and accountability to facilitate sufficient amelioration of dietary restriction. To overcome the aforementioned limitations inherent in the standard CBT treatment delivery approach to address fluctuations in dietary restriction, we developed an intervention system called SenseSupport to augment standard CBT for binge eating. SenseSupport is a state-of-the-art intervention system that combines passive sensing technologies for continuous and unobtrusive collection of data on eating behaviors and analysis to detect dietary restriction with a just-in-time, adaptive intervention (JITAI) system to intervene in this behavior in real time. SenseSupport has 3 key capabilities.

First, SenseSupport uses continuous glucose monitoring (CGM; ie, continuous and passive measurement of blood glucose levels) device to detect dietary restriction in real time. CGM can detect the within-day fluctuations in the dietary restriction because meal intake is associated with characteristic patterns of changes in blood glucose levels [35-40]. When individuals are not eating, their blood glucose levels are maintained at remarkably constant levels. In response to a meal, glucose levels fluctuate, with simple carbohydrates producing a quick and distinct rise in glucose and meals heavy in protein, fat, or fiber producing slower, less steep, and longer-lasting increases in glucose [41-43]. SenseSupport transfers the real-time CGM data to a smartphone and analyzes these data using an embedded meal detection algorithm to accurately detect meal consumption and estimate the size and macronutrient content of a meal. The meal detection app is based on the parameter-invariant algorithm developed by Weimer et al [40] in 2016. The parameter-invariant algorithm is *invariant* to individual physiological parameters and therefore achieves near-constant accuracy across the population without individual tuning (as required in previous algorithms).

Second, SenseSupport uses a JITAI system (a smartphone app–based system that uses real-time analysis of data to deliver momentary interventions at identified times of need) that delivers real-time brief CBT-based interventions when dietary restriction and other ED symptoms, including binge eating and purging, are detected. These interventions are designed to provide in-the-moment reminders to patients to eat regularly as they go about their daily lives, thereby augmenting the therapeutic content delivered in-session during CBT. These reminders are hypothesized to improve treatment outcomes as patients often report engaging in habitual dietary restriction, of which they may not be consciously aware, during treatment, and increased awareness of problematic behavior is essential to changing one’s behavior. For example, when the meal detection algorithm detects fasting behavior, SenseSupport’s JITAI system delivers an intervention encouraging patients to eat regularly throughout the day to prevent future binge episodes or suggesting use of problem-solving skills to address barriers to regular eating. JITAIs as augmentation of treatment-as-usual have demonstrated promise for improving outcomes for a variety of mental health conditions, including substance use disorders, schizophrenia, and affective disorders [44].

Third, SenseSupport includes a clinician portal that displays objective data on dietary restriction and ED behaviors from the CGM data to the treating clinician. Clinicians can quickly and easily view patients’ eating behaviors and use this information to guide treatment planning and implementation. For example, if clinicians observe continued engagement in dietary restriction between sessions, they may have an in-depth discussion to spur motivation to reduce restrictive eating and encourage the between-session practice of regular eating. The objective and real-time detection of dietary restriction from CGM data also allows for accurate, less burdensome, and more complete data collection during standard CBT for binge eating to further guide therapeutic work during CBT.

In the remainder of this paper, we present data from a small open clinical trial (N=30) in which patients with clinically significant binge eating and restrictive eating received 12 weeks of CBT treatment and completed electronic self-monitoring of eating and ED behaviors on a smartphone app (the SenseSupport app). The SenseSupport app also collected and analyzed the CGM data using a meal detection algorithm. The app had two additional features that could be turned on and off: (1) sharing CGM data with the study clinicians and (2) a JITAI system to detect and intervene in dietary restriction. We used an ABAB design (A=CGM data sharing and JITAIs-Off, B=CGM data sharing and JITAIs-On) to test the feasibility, acceptability, and target engagement of SenseSupport when paired with a CBT treatment program. The primary aims of this study were (1) to test the hypothesis that SenseSupport will be a feasible and acceptable system for use during a 12-week CBT treatment protocol; (2) to test the hypothesis that larger decreases in dietary restriction (ie, episodes of fasting for ≥5 hours, limiting the overall amount of food consumed, number of days when ≥8 hours of fasting was observed, excluding specific foods, following specific dietary rules, and desire for empty stomach) will be observed during JITAIs-On phases compared with JITAIs-Off phases; (3) to test the hypothesis that CBT augmented by the SenseSupport system will yield large decreases in binge eating, compensatory behaviors, and global eating pathology in pre- to posttreatment results; and (4) to test the hypothesis that larger decreases in binge eating and compensatory behaviors will be observed during JITAIs-On phases compared with JITAIs-Off phases.

## Methods

### Participants

Participants were recruited through professional referrals and radio, newspaper, and web-based (social media) advertisements. Advertisements called for individuals who engaged in fasting and binge eating to participate in clinical treatment studies. Participants included adults who met the Diagnostic and Statistical Manual of Mental Disorders, Fifth Edition criteria for BED or BN (ie, BED: experienced at least 12 episodes of binge eating in the past 3 months, accompanied by marked distress about binge eating and at least three binge eating features [eating rapidly; eating until excessively full; eating large amounts of food in the absence of hunger; eating alone because of embarrassment; or feeling disgusted, depressed, or very guilty]; BN: experienced at least 12 episodes of binge eating and at least 12 episodes of inappropriate compensatory behavior in the past 3 months, accompanied by excessive influence of body shape and weight on self-evaluation) and had 3 or more episodes of fasting for >5 waking hours per week in the last 4 weeks. Individuals were excluded if they (1) were receiving treatment for an ED or behavioral weight loss, (2) required immediate treatment for medical complications because of the ED, (3) were experiencing other severe psychopathology that would limit the participants’ ability to comply with this study (eg, severe depression with suicidal intent), (4) were not stable on psychiatric medications for at least 1 month, (5) had diabetes, (6) were taking a medication known to impact insulin or glucose levels, (7) had a history of bariatric surgery, (8) were pregnant or nursing, or (9) had a BMI <17.5 or >40.

### Procedure

Participants attended a Zoom-based baseline assessment in which they completed the Eating Disorder Examination (EDE) and self-report questionnaires (demographics and BMI) to confirm eligibility. Upon eligibility, participants were sent a Dexcom G6 CGM and a MiFit smart band for tracking activity, heart rate, and sleep. They were asked to trial the devices for 3 days before committing to the study for 12 weeks. The participants met the research coordinator via Zoom videoconference to arrange the trial period. They were instructed on how to download and use the SenseSupport phone app and MiFit smart band app. They were also instructed on how to use the Dexcom G6 CGM system. Participants were told to monitor all instances of food intake, ED behaviors, and mood on the SenseSupport app as close to real time as possible. The trial period occurred immediately following the baseline assessment. After completing the trial period and agreeing to commit to the study obligations, participants were sent additional CGM sensors and transmitters to last the 12 weeks of the study.

After the trial period, the participants began 12 weeks of therapy sessions. Participants completed telehealth treatment sessions with a study therapist once a week. In weeks 1 to 2, participants wore the CGM devices and tracked their eating behaviors and mood in the SenseSupport app but did not receive JITAIs, and therapists did not have access to the clinician portal (eg, an *A* period of the ABAB design). To track eating behaviors, including ED behaviors and mood, participants opened the smartphone app, initiated an entry, and answered a series of questions related to their eating behavior, including the type of eating episode, the context (ie, time and location) of eating episodes, and whether loss of control was experienced and the participant engaged in compensatory behaviors. In the same entry, participants rated their current experience of anxiety or worry, sadness or depression, and other emotions. Throughout the study, participants could view previous entries within the SenseSupport app. During weeks 3 to 4, JITAIs were *turned on* such that patients began to receive push notifications based on CGM data, and therapists had access to the clinician portal and were instructed to review CGM data during their session with patients (eg, a *B* period of the ABAB design). The A/B periods were repeated (weeks 5-6, and 9-10 were *A* periods; weeks 7-8, and 11-12 were *B* periods) to test whether the amelioration of dietary restriction was due to the use of the SenseSupport and not simply to the effect of time in treatment. Weekly data collected at each therapy session included the therapists’ and participants’ weekly questionnaires on issues reported with the CGM or SenseSupport app, a modified version of the EDE Questionnaire (EDE-Q) restraint subscale that asked about the preceding 7-day period (rather than the usual 28-day period), weekly participant-reported episodes of fasting for ≥5 hours, and finally, weekly participant-reported ED behaviors (binge episodes and compensatory behaviors).

Assessments of feasibility, acceptability, target engagement, and clinical outcomes were completed between sessions 4 and 5, between sessions 8 and 9, and after session 12 (posttreatment assessment) by the study team. Feasibility data included CGM data, weekly therapist and participant questionnaires, and retention calculations. Acceptability data included participant feedback and questionnaires. Target engagement data included the full EDE, the modified EDE-Q restraint subscale, participant-reported episodes of fasting for ≥5 hours, and participant-reported ED behaviors.

### Ethics Approval

This study was approved by the Institutional Review Board of Drexel University (protocol number 1907007293). Informed consent was obtained from all the participants before any study procedures.

### Measures

#### Baseline Characteristics

##### Demographics Questionnaire

The participants were asked to provide information such as age, sex, ethnicity, and socioeconomic status.

##### BMI Measures

Height and weight data were collected before the treatment was started and at each assessment point.

#### Feasibility

##### Retention

To assess feasibility, the percentage of participants enrolled in this study retained at each assessment point was collected. We also examined the percentage of participants who completed the 3-day trial period but declined to continue participation in the study based on this trial.

##### CGM Data

The Dexcom G6 CGM sensor provided blood glucose readings (in mg/dL) every 5 minutes. Participants wore CGM sensors, which used 0.5-inch flexible wires inserted into the abdomen to collect blood glucose readings. A transmitter attached to the sensor wirelessly transmitted the blood glucose readings to the SenseSupport app portal. The percentage of total expected CGM data that were collected was computed for each participant as a measure of the feasibility of the SenseSupport system.

##### Weekly Therapist and Participant Questionnaires

Therapists and participants reported problems with the CGM sensors and the SenseSupport app in web-based questionnaires at each therapy session.

#### Acceptability

##### Participant Feedback Questionnaire

Participants completed a feedback questionnaire at session 12, which was used to obtain qualitative ratings regarding the acceptability of and compliance with the SenseSupport system. The feedback questionnaire included questions about the comfort, pain, disruption of daily life, ease of use, and helpfulness of the SenseSupport system.

##### Participant Feedback Interviews

Participants completed interviews after sessions 4, 8, and 12 to provide qualitative feedback regarding their experiences with the SenseSupport system.

#### Target Engagement

##### EDE-Q Restraint Subscale

The EDE-Q is a self-report version of the EDE. The restraint subscale consists of 5 items that measure dietary restraint, including overall dietary restraint, avoidance of eating for ≥8 hours, desire for an empty stomach, food avoidance, and dietary rules. Participants completed each of these items, which were modified to ask about the past 7 days (rather than the past 28 days) at each therapy session. These items were examined as measures of dietary restraint (ie, attempts to limit food consumption).

##### Weekly Participant-Reported Episodes Fasting for ≥5 Hours

Participants reported the frequency with which they went ≥5 hours without eating anything over the past 7 days at each therapy session. This was examined as a measure of dietary restriction (ie, the actual limitation of food consumption).

#### Treatment Outcomes

##### EDE-Measured ED Symptoms

The EDE [12] is a semistructured clinician-administered interview that includes 4 subscales (restraint, eating concern, weight concern, and shape concern). Treatment outcomes were assessed through reductions in loss-of-control episodes, compensatory behaviors, and global EDE severity scores. The EDE global score can range from 1 to 5, with a higher number indicating a more severe pathology. The score was calculated by summing the subscales and dividing the total score by 4. A score of 4 or higher was considered clinically significant. A shortened version of the interview was conducted between sessions 4 and 5 and sessions 8 and 9, while the full interview was administered at baseline and after session 12. The shortened interview ended after the compensatory behaviors section of the EDE (the shape and weight concerns sections were excluded).

##### Weekly Participant-Reported ED Behaviors

At each therapy session, participants reported the number of episodes of binge eating and compensatory behaviors in which they engaged over the previous 7 days (eg, “In the past 7 days, how many binge episodes did you have?” and “In the past 7 days, how many times did you vomit to compensate for a binge episode?”).

### Statistical Analyses

#### Feasibility

Feasibility was characterized using percent retention during treatment. We considered retention ≥80% following the 3-day trial period and at each assessment point to be adequate feasibility. The percentage of data obtained from the CGM sensors was also quantified, with ≥80% of the expected data being considered adequate. Finally, the issues reported with the SenseSupport system by therapists and patients were also summarized.

#### Acceptability

Acceptability was characterized using patient-reported acceptability ratings at session 12, with >80% ratings of 4 or 5 (on 1 to 5 scales wherein higher scores indicate greater acceptability) and with >80% ratings of 1 or 2 (on 1 to 5 scales wherein lower scores indicate greater acceptability) being considered acceptable.

#### Target Engagement

Target engagement variables included participant-reported episodes of fasting for ≥5 hours, and responses to the EDE-Q restraint subscale items measured each week of treatment. The slope of change in target engagement variables (weekly episodes of fasting for ≥5 hours, days of limiting overall amount consumed, number of days when ≥8 hours of fasting was observed, days excluding foods from diet, days following specific dietary rules, and days with desire for empty stomach) was computed for each *A* period and *B* period. Consecutive *A* periods and *B* periods were paired and compared using 1-tailed paired samples *t* tests for each variable of interest (ie, the period from session to 1-3 was compared with the period from session 3-5; the period from session 5-7 was compared with the period from session 7-9).

#### Treatment Outcomes

Treatment outcomes (past month binge episodes; compensatory behaviors; and EDE global and subscale scores, which was measured by the EDE) were assessed by comparing pre- and posttreatment measures using paired sample 1-tailed *t* tests. In addition, slopes of change in treatment outcome variables (weekly binge episodes and weekly compensatory behaviors) were computed for each *A* period and B period. Consecutive *A* and *B* periods were paired and then compared via paired samples *t* tests.

## Results

### Overview

Demographic information is shown in Table 1. The feasibility, acceptability, treatment outcomes, and target engagement results are summarized in Table 2, Table 3, Table 4, and Table 5, respectively.

**Table 1:** Participant demographics (N=30).

		Participants
**Gender, n (%)**		
	Man	3 (10)
	Woman	26 (87)
	Genderqueer or gender nonconforming	1 (3)
**Race, n (%)**		
	White	27 (90)
	Black or African American	3 (10)
	Asian	2 (7)
	More than one race	2 (7)
**Ethnicity, n (%)**		
	Non-Hispanic or Latino	28 (93)
	Hispanic or Latino	2 (7)
**Baseline diagnosis, n (%)**		
	BN^a^	20 (67)
	BED^b^	10 (33)
**Employment status^c^, n (%)**		
	Full time	17 (57)
	Part time	6 (20)
	Full-time student	4 (13)
	Part-time student	1 (3)
	Disability or social security	1 (3)
	No income	6 (20)
**Relationship status, n (%)**		
	Married	8 (27)
	Divorced	4 (13)
	Single	12 (40)
	Living with partner, but not married	3 (10)
	In relationship but not living with partner	2 (7)
	Widowed	1 (3)
**Household income (US $), n (%)**		
	0-10,000	1 (3)
	10,000-24,999	1 (3)
	25,000-34,999	1 (3)
	35,000-49,999	6 (20)
	50,000-74,999	6 (20)
	75,000-99,999	3 (10)
	≥100,000	10 (33)
	Unknown	2 (7)
Age (years), mean (SD)		37.10 (12.19)
BMI (kg/m^2^), mean (SD)		29.51 (5.20)

^a^BN: bulimia nervosa.

^b^BED: binge eating disorder*.*

^c^For employment status, if a participant indicated that they were a student while also working, both responses would be reflected in the data above.

**Table 2:** Feasibility of SenseSupport.

					Values
**Retention (n=30), n (%)**					
	**Time of assessment**				
		Week 4	29 (96.7)		
		Week 8	27 (90)		
		Posttreatment	25 (83.3)		
**Reported problems with SenseSupport app (n=333 therapy sessions), n (%)**					
	By participants			140 (42)	
	By therapists			48 (14.4)	
**Reported problems with CGM^a^ sensors (n=333 therapy sessions), n (%)**					
	By participants			16 (4.8)	
	By therapists			7 (2.1)	
Compliance with CGM sensors (%), mean (SD; range)					67.4 (18.3; 16.8-92.2)

^a^CGM: continuous glucose monitoring.

**Table 3:** Acceptability of SenseSupport (n=24).

	Values, mean (SD; range)	Value, n (%)
In the past 4 weeks, on average, how comfortable did you feel wearing the CGM^a^ sensor? (1=Completely uncomfortable to 5=Completely comfortable)	4.50 (0.93; 2-5)	19 (79) rated 4 or 5
In the past 4 weeks, on average, how painful was the insertion of the CGM sensor? (1=Not at all painful to 5=Extremely painful)	1.38 (0.71; 1-4)	23 (96) rated 1 or 2
How much did the CGM sensor disrupt your daily life? (1=It was not at all disruptive to 5=It was extremely disruptive)	1.33 (0.48; 1-2)	24 (100) rated 1 or 2
In the past 4 weeks, on average, how easy was it to learn how to navigate the full SenseSupport system? (1=Not at all easy to learn to 5=Extremely easy to learn)	4.58 (0.65; 3-5)	22 (92) rated 4 or 5
How helpful were the push notifications? (1=Not at all helpful to 5=Extremely helpful)	4.74 (0.45; 4-5)	24 (100) rated 4 or 5
How much did you like using the SenseSupport system with your therapist? (1=Not at all to 5=Extremely; n=6)	4.00 (0.63; 3-5)	6 (83) rated 4 or 5
Overall, how likely would you be to use the SenseSupport system in the future as part of treatment for an eating disorder? (1=Not at all likely to 5=Extremely likely)	4.08 (0.83; 2-5)	19 (79) rated 4 or 5
Overall, how likely would you be to recommend the SenseSupport system in the future as part of treatment for an eating disorder? (1=Not at all likely to recommend to 5=Extremely likely to recommend)	4.21 (1.02; 1-5)	19 (79) rated 4 or 5

^a^CGM: continuous glucose monitoring.

**Table 4:** Treatment outcomes of SenseSupport^a^.

	Baseline, mean (SD)	Posttreatment, mean (SD)	Slope of change during data sharing- and JITAIs^b^-On weeks, mean (SD)	Slope of change during data sharing- and JITAIs-Off weeks, mean (SD)	*t* test (*df*)	*P* value^c^	Cohen *d*
Past month total binge episodes	23.88 (11.23)	4.60 (6.25)	N/A^d^	N/A	10.36 (24)	*<.001*	2.07
Past month total compensatory behaviors	15.96 (19.41)	3.60 (14.87)	N/A	N/A	3.40 (24)	*.001*	0.68
EDE^e^ restraint subscale score	2.37 (1.38)	0.93 (1.40)	N/A	N/A	4.34 (24)	*<.001*	0.87
EDE eating concern subscale score	1.97 (1.10)	0.97 (1.20)	N/A	N/A	3.43 (24)	*.001*	0.69
EDE weight concern subscale score	3.75 (1.01)	2.08 (1.22)	N/A	N/A	6.14 (24)	*<.001*	1.23
EDE shape concern subscale score	4.22 (0.87)	2.79 (1.14)	N/A	N/A	5.83 (24)	*<.001*	1.17
EDE global score	3.08 (0.76)	1.69 (1.10)	N/A	N/A	6.25 (24)	*<.001*	1.25
Weekly binge eating episodes	N/A	N/A	0.06 (.11)	0.03 (.15)	0.73 (24)	.23	0.11
Weekly total compensatory behaviors	N/A	N/A	−.01 (.16)	0.06 (.23)	1.78 (24)	*.04*	0.25

^a^Positive values for change in eating disorder behaviors indicate decreases in the frequency of behavior during the period of interest.

^b^JITAI: just-in-time, adaptive intervention.

^c^Italicized *P* values indicate statistical significance.

^d^N/A: not applicable.

^e^EDE: Eating Disorder Examination.

**Table 5:** Target engagement of SenseSupport^a^.

	Slope of change during data sharing- and JITAIs^b^-On weeks, mean (SD)	Slope of change during data sharing- and JITAIs-Off weeks, mean (SD)	*t* test (*df*)	*P* value^c^	Cohen *d*
Weekly episodes of fasting for ≥5 hours	0.04 (0.14)	0.07 (0.20)	−0.82 (44)	.21	−0.13
On how many days have you been deliberately trying to limit the amount of food you eat to influence your shape or weight (whether or not you have succeeded)?	0.03 (0.08)	−.01 (0.10)	1.60 (44)	.06	0.24
On how many days have you gone for long periods (8 waking hours or more) without eating anything at all to influence your shape or weight?	0.01 (0.06)	0.02 (0.08)	−1.05 (44)	.15	−0.16
On how many days have you tried to exclude from your diet any foods that you like to influence your shape or weight (whether or not you have succeeded)?	−0.003 (0.11)	.02 (0.11)	−0.84 (44)	.20	−0.13
On how many days have you tried to follow definite rules regarding your eating (eg, a calorie limit) to influence your shape or weight (whether or not you have succeeded)?	0.02 (0.11)	0.01 (0.08)	0.77 (44)	.22	0.12
On how many days have you had a definite desire to have an empty stomach with the aim of influencing your shape or weight?	0.04 (0.09)	−0.002 (0.08)	1.69 (44)	*.049*	0.25

^a^Positive values for change in eating disorder behaviors indicate decreases in the frequency of behavior during the period of interest.

^b^JITAI: just-in-time, adaptive intervention.

^c^Italicized *P* values indicate statistical significance.

### Feasibility

The participants’ retention was high in this study. Only 3% (1/30) of participants completed the 3-day trial period and then declined to participate in the study (constituting 3.2% of the sample who completed the trial period). Furthermore, 83% (25/30) of the participants completed all treatment sessions and assessments. The mean percentage of expected CGM data that were collected was 67.4%, and the range was broad (16.8%-92.2%). Notably, only 10 participants (33% of the sample) met the threshold for the successful collection of >80% of the expected CGM data. Participants (28/30, 93%) and therapists (7/8, 88%) also frequently reported app-related problems, including frequent disconnections (reported by patients at 123 sessions and therapists at 35 sessions), nondelivery of JITAIs when expected (reported by patients at 5 sessions and therapists at 5 sessions), or delivery of JITAIs without the occurrence of ≥5 hours without eating (reported by a patient at 1 session), slow loading of app content (reported by a patient at 1 session), unspecified bugs (reported by patients at 10 sessions and therapists at 4 sessions), difficulty connecting new sensors to the app (reported by patients at 3 sessions and a therapist at 1 session), and failure to save electronic self-monitoring records (reported by patients at 5 sessions and therapists at 5 sessions). Participants (11/30, 37%) and therapists (2/8, 25%) also reported issues with the sensors, including sensor insertion being more uncomfortable than usual (reported by patients at 2 sessions), location of the sensor relative to clothing impacting data collection (reported by a patient at 1 session and a therapist at 1 session), sensors falling off (reported by a patient at 1 session), and nonspecific sensor or transmitter failures (reported by patients at 11 sessions and by therapists at 3 sessions).

### Acceptability

Participants rated the use of the SenseSupport system as highly acceptable. Of the 24 participants with complete acceptability data, 19 (79%) and 24 (100%) participants rated wearing the CGM sensors as comfortable and minimally disruptive to their lives, respectively. More than 95% (23/24) of the participants rated the insertion of the CGM sensors as minimally painful. The participants also reported that the SenseSupport system was easy to learn (22/24, 92%) and useful (24/24, 100%). Approximately 83% (5/6; this question had substantial missing data) of participants also reported that they liked using the system, and 79% (19/24) reported that they would use the system or would recommend the system to others as part of ED treatment. Participants reported that they enjoyed the supportive accountability provided by their clinician’s ability to view their glucose levels, the reminders to eat regularly when JITAIs were turned on, and the opportunity to observe how their bodies processed meals and snacks they had consumed (refer to Textbox 1 for more details). The established benchmarks for acceptability were met (>80% ratings of >4 or 5 and >80% ratings of <1 or 2, depending on the question) for insertion pain, disruptiveness, ease of learning the system, helpfulness of push notifications, and enjoyment of using the system (see Table 3 for mean ratings). However, the a priori benchmarks were not met for the likelihood of using the system in future ED treatment or the likelihood of recommending the system to others for use during ED treatment.

Qualitative participant feedback particularly emphasized the ease of using the SenseSupport system and CGM sensors, the unobtrusiveness of the CGM sensors, the benefits of supportive accountability facilitated by observable patterns in blood glucose associated with meals, the helpful push notifications reminding them to eat regularly, and an appreciation of learning about their physiological responses to eating (Textbox 1). Several participants endorsed that they wished that they had received more push notifications to remind them to eat regularly.

**Qualitative participant feedback on continuous glucose monitoring (CGM) sensors and SenseSupport system.**

**Unobtrusiveness of CGM sensors**

- “I like that [the CGM] is so small so you can’t really see it through my clothing.”
- “I didn’t really notice [the CGM] because it was small.”
- “There wasn’t any pain. [The CGM] didn’t interfere with activities.”
- “[I liked that the CGM] is relatively discrete, it doesn’t come off, it sticks for the most part. The insertion is easy.”
- “[The CGM] is very easy to put on and forget it’s there.”

**Ease of use of SenseSupport system**

- “I thought [SenseSupport] was pretty easy to use.”
- “It was easy to use and understand.”
- “It was pretty easy to navigate.”
- “I thought that [the SenseSupport system] was really easy to use and I found it really helpful.”
- “[The SenseSupport system] was super simple.”

**Supportive accountability**

- “It helped keep me accountable to what I was recording. There was data to match up with what I was recording.”
- “It made me more likely to stick to regular eating and avoid binging because I knew what the glucose levels would show up as and I wanted that to look good.”
- “There was no lying with the glucose monitoring. I knew if it was going in my mouth it was going on paper.”
- “[The system] gives you accountability because your therapist is also using your portal to monitor your treatment.”
- “[The system] helps you see your eating patterns and just create better eating habits. It helps you 24 hours of the day. There’s always an app helping you. It’s the hardest afterwards now there’s nobody watching. Going from 100% to 0% accountability post-treatment is hard.”
- “I liked that [my therapist] could see what I was eating and noticed changes I couldn’t notice. The glucose graphs were really interesting.”
- “[Using SenseSupport] was very productive and very positive. [I liked] the external structure and accountability that came with the system as a whole.”
- “I liked the involved process and self-checks to have accountability with and without my therapist. [The SenseSupport system] made me check myself to eat regularly. [There was] a lot more consistency with all the parts operating together.”

**Push notifications as reminders to eat regularly**

- “[The push notifications] were encouraging in the moment.”
- “[The notifications] would come when I didn’t eat frequently. They were helpful. I wish there were more.”
- “If [the SenseSupport system] felt like I had loss of control or overeating, it helped me get back to the structure that we had been on previously.”
- “I thought [the reminder push notifications] were really helpful.”
- “[The notifications] would remind me when I wasn’t eating. That was good.”

**Learning about physiological responses to food**

- “It was helpful to be able to record my food and have [my therapist] tell me that what I was eating wasn’t adequate according to my blood glucose levels and whether or not I should continue eating like that.”
- “[The system] shows you how your body responds to things and made me want to eat regularly so I can elicit a specific response from my body...I believe my metabolism is all messed up. I spent a lot of time fasting and not eating but seeing how my body responds to food made me more comfortable with food.”
- “I really liked having the specific information about checking glucose levels and comparing what I was eating and when.”
- “[SenseSupport] was helpful because it helped me to keep consistently eating throughout the day and making sure to eat enough at each meal. It helped me align my hunger cues more and organize, get into a routine.”
- “[SenseSupport] helped keep me accountable and connect what I was eating to my body.”
- “It helped to understand the importance of regular eating and what a normal glucose pattern should look like versus what my pattern looked like.”
- “I really enjoyed going over the glucose data with [my therapist]. Seeing all of that really helped me want to regulate my body. [It] made me want to eat for my body’s wellbeing.”

### Target Engagement

The frequency of desiring an empty stomach to influence shape or weight decreased significantly more rapidly during JITAIs-On weeks compared with JITAIs-Off weeks with a small effect size. There was also a trend-level effect for the frequency of deliberately trying to limit the amount of food consumed to influence shape or weight, with greater decreases during periods during the JITAIs-On weeks. There were no significant differences among episodes of fasting for ≥5 hours, going long periods without eating, excluding foods from diet, or following definite rules regarding eating by notification delivery.

### Treatment Outcomes

Participants showed significant decreases in the frequency of binge eating and compensatory behaviors, and in the severity of eating pathology from pre- to posttreatment with medium (compensatory behaviors and EDE eating concern subscale scores) and large (binge eating episodes; EDE restraint, weight concern, and shape concern subscale scores; and EDE global scores) effect sizes. Contrary to the hypotheses, the change in the frequency of weekly compensatory behaviors was significantly more rapid during JITAIs-Off periods compared with JITAIs-On. Changes in the frequency of weekly binge eating episodes were not significantly different during JITAIs-On weeks compared with JITAIs-Off weeks.

## Discussion

This study is the first to evaluate a novel intervention system that uses CGM sensors to augment CBT for binge eating to improve dietary restriction and treatment outcomes.

### Principal Findings

The high retention rates for the study assessments and sessions demonstrated the feasibility of the SenseSupport system. However, the benchmarks for successful data collection from CGM were not met. The most notable barrier to achieving this benchmark was the connectivity issues between the CGM sensors and the SenseSupport app owing to technical problems in the software development kit provided by Dexcom during the study. Because the publicly available application programming interface for Dexcom devices has a built-in 3-hour time delay and thus would not provide data quickly enough for use in the current system, we relied on the donated software development kit for this initial proof-of-concept development phase. These findings may suggest that although it may be feasible to use SenseSupport as an augmentation to in-person CBT, future development work is needed to ensure that technological problems owing to Bluetooth connection dropping between the CGM sensor and the SenseSupport system are resolved before additional testing is indicated. Future iterations of SenseSupport may involve either using newer-generation CGM sensors that may better integrate with the SenseSupport app or enabling the software development kit from Dexcom to support more robust data collection.

Despite the concerns regarding missing CGM data, SenseSupport demonstrated high acceptability and achieved nearly all a priori benchmarks for the acceptability of the system. Qualitatively, the patients indicated that the CGM sensors were comfortable to wear and minimally disruptive to their lives. A majority of patients perceived SenseSupport to be useful in learning about patterns in blood glucose associated with meals and reminding them to eat regularly, and they expressed a desire to receive more frequent JITAIs during treatment. SenseSupport fell marginally short of meeting the benchmarks for the likelihood of using or recommending the system to others as part of the ED treatments. When probed further, this appeared to be largely due to the burdensome nature of manually reconnecting the sensor and app after the Bluetooth connections failed, as discussed earlier. These interruptions may have reduced the perceived value of SenseSupport to augment ED treatments despite the participants rating the overall SenseSupport system favorably.

Compared with JITAIs-Off weeks, JITAIs-On weeks had small effect size improvements in desire for empty stomach (ie, intent that maintains dietary restriction) and reductions in the frequency of deliberately trying to limit the amount of food consumed (ie, actual restriction) to influence shape or weight at the trend-level, suggesting the possible benefit of the SenseSupport system in reducing both intent and actual dietary restriction. However, contrary to the hypotheses, the observed differences among episodes of fasting for ≥5 hours, going long periods without eating, excluding foods from diet, or following definite rules regarding eating during JITAIs-On compared with JITAIs-Off weeks were small. One possible reason for the small effect size differences between *A* and *B* periods may be that during the full duration of the study, participants were receiving CBT treatment with a specific focus on reducing dietary restriction. In other words, it may be plausible that the changes through CBT treatment were so notable that there was little opportunity for the JITAIs to have a significant impact on dietary restriction. In addition, in *A* and *B* periods, participants continued to wear the sensors and track eating behavior and ED symptoms in the SenseSupport app. Thus, it is possible that the high levels of overlap between the JITAI-On and JITAI-Off weeks made it difficult to observe differences between the conditions. In addition, a common challenge of ABAB study designs is that carryover effects may be observed that extend beyond the periods being measured. For example, in this study, it is possible that JITAIs delivered in *B* weeks led to general amelioration of dietary restriction that persisted into the next *A* period. Future research using alternative study designs is needed to determine the additive value of the SenseSupport system above and beyond CBT.

We observed medium effect size improvements in the frequency of compensatory episodes and large effect size improvements in binge eating frequency and cognitive symptoms of ED pathology in pre- to posttreatment results. However, contrary to the hypotheses, the reduction in the frequency of binge eating during JITAIs-On weeks was only marginally higher than the improvements in binge eating frequency during JITAIs-Off weeks, perhaps owing to many of the limitations described for detecting changes in our target engagement variables as a result of the ABAB design described earlier.

### Comparison With Prior Work

The medium effect size improvements observed in the frequency of compensatory episodes and large effect size improvements in binge eating frequency and cognitive symptoms of ED pathology in pre- to posttreatment results are notable when compared with those reported in meta-analyses and systematic reviews testing the efficacy of CBT for BN and BED (ie, an average small effect size improvement in compensatory behaviors and an average medium size improvement in cognitive symptoms) [45]. The strong outcomes observed over the full duration of treatment suggest the promise of the SenseSupport system as an augmentation of CBT for EDs characterized by binge eating and indicate that additional research is warranted. Although these results are preliminary and replication is needed in large-scale clinical trials, the SenseSupport system may also be a useful augmentation for other EDs, such as anorexia nervosa, where individuals are at risk of overreporting their eating episodes and could benefit from receiving a treatment that uses the SenseSupport system to collect objective data on dietary restriction and ED behaviors to guide treatment planning and implementation. In addition, SenseSupport system could also augment treatments for comorbid medical conditions with binge eating that are associated with high glucose variability. For example, the SenseSupport system could provide objective data on blood glucose levels in individuals with comorbid type 2 diabetes mellitus and binge eating to guide their eating-related decisions on a day-to-day basis that may further help stabilize blood sugar levels.

### Limitations

This study has several limitations. First, we used an ABAB design, which may have introduced the carryover effects of JITAIs-On weeks to JITAIs-Off weeks. Thus, this design substantially reduces our confidence in the causal effect of JITAIs in improving dietary restriction or behavioral clinical outcomes such as binge eating and compensatory behaviors. A randomized controlled trial is the logical next step in determining whether SenseSupport is the causal factor in improving dietary restriction and eating pathology. Second, it is possible that the participants’ eating decisions were influenced merely by wearing CGMs, in addition to those guided by the SenseSupport app and interventions, as reactivity to these devices has been documented in other populations [46]. Our inability to clearly delineate the effects of the CGM sensors from those of SenseSupport interventions is a limitation of the study although the effects of wearing CGMs could partially explain the changes in eating behavior observed during JITAIs-Off periods. Similarly, the participants were compensated for the completion of the assessments and received free treatment by participating in the study. These incentives may have influenced the participants’ engagement in the study, thereby affecting the feasibility data collected. Third, the study did not include a follow-up assessment that precluded the knowledge of the effect of SenseSupport-augmented CBT in maintaining long-term gains in dietary restriction and eating pathology. Future research would benefit from assessing the long-term effects of SenseSupport-augmented CBT for binge eating. Fourth, in this study, the treatment included multiple intervention components (eg, digital self-monitoring, CGM data sharing with study clinicians, and the JITAI system), which precluded the understanding of the unique contribution of CGM data sharing with clinicians and the JITAI system as an augmentation of CBT treatment. Future research should attempt to isolate these different technological intervention components that may impact dietary restriction and clinical outcomes, as well as test the use of JITAIs to deliver more complete CBT interventions as an augmentation of CBT. Fifth, our methods preclude determining the percentage of missing CGM data that was due to CGM app malfunctioning. Sixth, similar to the previous ED studies, most patients were White women. Future research should attempt to replicate our findings in samples that are more diverse in race, ethnicity, and gender.

### Conclusions

In summary, this study successfully developed and deployed the first ever intervention system combining passive CGM sensors and the JITAI system as an augmentation of CBT to improve dietary restriction and clinical outcomes in EDs characterized by binge eating. Our findings suggest that the SenseSupport system is worthy of additional study in future through fully powered clinical trials.

## Abbreviations

ABAB: A, CGM data sharing and JITAIs-Off; B, CGM data sharing and JITAIs-On
BED: binge eating disorder
BN: bulimia nervosa
CBT: cognitive behavioral therapy
CGM: continuous glucose monitoring
ED: eating disorder
EDE: Eating Disorder Examination
EDE-Q: Eating Disorder Examination Questionnaire
JITAI: just-in-time, adaptive intervention

## Data Availability

Data from this project will be available upon reasonable request.
